# Bond Stress–Slip Model of BFRP Grid to ECC

**DOI:** 10.3390/ma15227965

**Published:** 2022-11-11

**Authors:** Langni Deng, Taisheng Li, Mengjun Zhong, Ling Liao, Hua Li

**Affiliations:** School of Civil Engineering and Architecture, Guangxi University of Science and Technology, Liuzhou 545006, China

**Keywords:** BFRP grid, ECC, composite materials, bond–slip, constitutive model, anchorage length

## Abstract

The bonding performance between a basalt fiber-reinforced composite material (BFRP) grid and an engineering cementitious composite (ECC) is the basis that affects the synergy between the two. However, the research on the bonding behavior between the FRP grid and ECC is limited; in particular, the theoretical study on the bond–slip intrinsic relationship model and a reliable anchorage length calculation equation is lacking. To study the bond–slip relationship between the BFRP grid and ECC material, we considered the parameters of BFRP grid thickness, anchorage length, ECC substrate protective layer thickness, and grid surface treatment, and conducted center pull-out tests on eight sets of specimens. By analyzing the characteristics of the bond–slip curve of the specimen, a bond–slip constitutive model between the BFRP grid and ECC was established. Combining the principle of equivalent strain energy, the calculation formula of the basic anchorage length of the BFRP grid in the ECC matrix was derived. Research shows that the bonding performance between the BFRP grid and ECC improves with the increase in the grid anchoring length, grid thickness, and ECC layer strength. Sand sticking on the surface of the BFRP grid can enhance the bonding force between the two. The established bond–slip constitutive model curve is in good agreement with the test curve. The bond–slip relationship between the BFRP grid and ECC can be described by the first two stages in the BPE model. The derived formula for calculating the basic anchorage length of the BFRP mesh in the ECC matrix is computationally verified to be reliable in prediction.

## 1. Introduction

Engineering cementitious composite (ECC) reinforced by chopped fibers, with excellent toughness and crack resistance [1,2], has become one of the preferred materials to effectively solve problems such as the development of concrete cracks [3]. However, the direction of load bearing by randomly distributed chopped fibers is not clear, which gives ECC the limitation of a low bearing capacity. Embedding a fiber-reinforced polymer (FRP) grid with strong tensile strength and corrosion resistance in the ECC matrix can effectively solve this problem [4]. Experimental studies have shown that, compared with ECC specimens, the uniaxial tensile failure mode of FRP grid-reinforced ECC composite specimens has changed from fiber pull-out or breakage to grid breakage, and its tensile properties have been significantly improved [5,6,7]. In addition, this composite material has shown good ductility and resistance to deformation and complex environmental erosion in material performance tests and tests of flexural and compressive bearing capacity of reinforced structures [8,9,10,11,12,13,14].

Further research on the bonding behavior, failure mechanism, and basic anchoring length calculation between the FRP grid and ECC is an important basis for effectively promoting the application of FRP grid-reinforced ECC composites in engineering. In the previous analysis and research on the bonding performance of steel or FRP bars and cement-based materials, researchers have conducted substantial research on the constitutive relationship of bonding and slippage and the influence parameters of bonding performance. Their methods and results are instructive for this paper. Mi et al. [15] considered the anchorage length, diameter, and shape of FRP bars to study the bonding performance between FRP bars and ECC. Studies have shown that the ultimate average bond stress of the specimen decreases with the increase in anchoring length or diameter, and the bond strength between threaded tendons and ECC is significantly higher than that of plain round tendons. Zhu et al. [16,17] found through drawing tests that the presence of the transverse steel strands of the steel strand grid can effectively slow down the relative slip between the steel strand grid and the ECC and improve the ductility of the specimen, and the bond–slip relationship model and the expression of the basic anchorage length established based on the experimental results and related studies had certain feasibility. Hossain [18] studied the bonding behavior between glass fiber-reinforced polymer (GFRP) bars and ECC through 90 beam specimens and proved that the bond strength predicted by the existing formula is conservative. Dalalbashi et al. [19] studied the bond–slip law between single and two-way FRP grids and a cement base and calculated the bond–slip relationship between the two through an iterative method. This provides a new idea for studying the bond–slip behavior of the FRP grid and ECC. In addition, some scholars have investigated the effect of different parameters on the bond–slip pattern between BFRP bars and materials, such as geopolymer concrete (GPC), and have derived relevant bond–slip models to describe it [20,21,22].

However, there are certain differences in the material structure and performance of FRP grids and FRP bars or steel bars. It is difficult to effectively describe the bonding behavior between FRP grids and ECC through the study of the bond performance between FRP bars or steel bars and ECC. Therefore, Jiang et al. [23] experimentally investigated the effects of factors such as embedding length and transverse mesh bundle of BFRP meshes on the bonding behavior of BFRP meshes to ECC and developed a bond–slip intrinsic relationship model considering the restraint of weft yarns, which still needs to consider more mesh configurations to work out more general conclusions. However, the current research on the bonding behavior between the FRP grid and ECC is quite limited, especially the theoretical research on the bond–slip constitutive relationship model and reliable anchor length calculation. The authors of [24] proposed a calculation method to convert the bond–slip curve between FRP reinforcement and the matrix into an equivalent linear model based on the principle of equivalent strain energy. Li et al. [25] established a unified linear cohesion model based on this method, deduced the calculation formula of the minimum anchorage length of FRP bars, and proved the correctness and feasibility of this method. It provides an important theoretical research foundation for the calculation of the basic anchorage length between the BFRP grid and ECC in this paper. In summary, it is necessary to further investigate the bonding and anchoring behavior of the FRP grid in ECC to establish a suitable bond–slip relationship intrinsic model and explore a reliable calculation method for the anchorage length of the FRP grid in ECC.

For this reason, in this paper, the BFRP grid thickness, anchorage length, ECC protective layer thickness, and grid surface treatment parameters were used as experimental variables to study the bonding performance between the BFRP grid and ECC. By analyzing the characteristics of the bond–slip curve of the specimen and combining the existing theoretical models at home and abroad, a constitutive model of the bond–slip between the BFRP mesh and the ECC was established. Combining the principle of equivalent strain energy, the calculation formula of the anchoring length of the BFRP grid in the ECC matrix was derived. We hope that the results of the study can provide some reference significance for research related to FRP grid-reinforced ECC composites.

## 2. Experimental Research

### 2.1. Test Materials

The main components of ECC used in the test and their mixing ratios are shown in Table 1. Among them, the quartz sand mesh number was 100–150, the water-reducing agent was a polycarboxylic acid superplasticizer, and the polyvinyl alcohol (PVA) fiber was produced by Japan Kuraray Company (Tokyo, Japan). The performance indicators are shown in Table 2. The measured cracking strengths of ECC1 and ECC2 were 3.6 and 4.8 MPa, the modulus of elasticity was 9.87 and 12.31 GPa, and the ultimate tensile strain was 4.1% and 3.2%, respectively.

The BFRP grid was produced by Jiangsu Green Material Valley Company (Nanjing, China), and the square mesh side length was 50 mm. Five samples of each of the three different thicknesses of BFRP grids were subjected to tensile tests, and the performance indexes of the BFRP grid materials are shown in Table 3.

### 2.2. Pull Test

**Figure 1:**
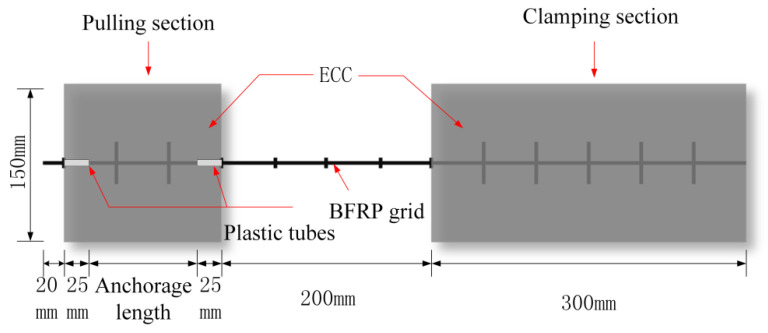
BFRP grid and ECC drawing sample design schematic diagram.

As shown in Table 4, the experiment considered different variables to design and produce 8 sets of 24 drawing specimens. To eliminate the end effect between the matrix and the grid, a layer comprising a 25 mm-long plastic thin tube was sleeved on the end of the BFRP grid embedded in the matrix of the drawing section. The specific design of the test piece is shown in Figure 1.

**Figure 2:**
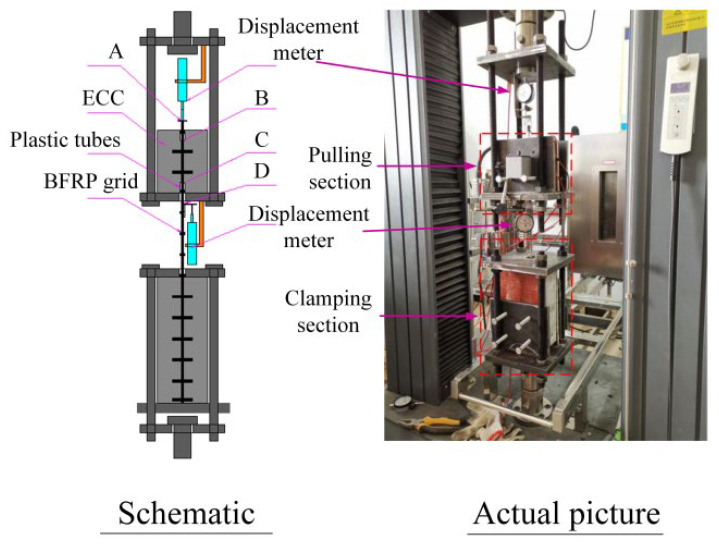
Drawing test device diagram.

The pull-out test device for the adhesion between the BFRP grid and ECC is shown in Figure 2. We fixed the pull head of the loading device on the ETM105D universal testing machine (Wanchen Testing Machine Co., Ltd., Jinan, China) with a pin and nut, and the loading rate was 0.2 mm/min.

See Table 4 for specific test variables and design information. Considering the influence of ECC strength on bond–slip performance, a group of ECC2 specimens with different strengths was set up for comparison with the ECC1 specimen group. Summarizing the experience of existing studies, we found that the anchorage length of the FRP grid, FRP grid thickness, and ECC protective layer thickness also had an effect on bond–slip performance in the reinforcement tests using FRP grid-reinforced ECC composites, so the conventional dimensional design in these studies was referred to in the parameter design of this paper.

To measure the slip of the BFRP grid, displacement meters were installed at points A and D of the grid. There was no deformation in section AB, so the displacement measured at points A and B was the same, but the deformation of section CD needed to be deducted, and the amount of deformation can be expressed as:

(1) $$\Delta S_{CD}=\frac{Pl_{CD}}{E_{f}A_{f}}$$

where P is the pulling force, $l_{CD}$ is the distance of the *CD* segment, $E_{f}$ is the elastic modulus of the grid, and $A_{f}$ is the cross-sectional area. Then, the actual displacement of the loading end, *C*, can be expressed as:

(2) $$S=S_{D}-\Delta S_{CD}$$

where $S_{D}$ is the actual displacement measured by the *D* point displacement meter.

The bond strength, τ, can be expressed as:

(3) $$\tau =\frac{P}{u_{f}l_{a}}$$

where $u_{f}$ is the perimeter of the BFRP grid section and $l_{a}$ is the bonding length of the grid.

### 2.3. Test Results and Analysis

There were two main forms of damage of the specimen: (1) the grid was pulled out or (2) the grid was broken. In the first scenario, with the increase in the drawing force, the horizontal grid bundle was stretched and squeezed, and the grid slipped obviously inside the ECC matrix. After the grid was pulled out, the inside of the ECC matrix was slightly worn, without splitting. In the second scenario, the grid suddenly broke after reaching the ultimate bearing capacity, and the overall bonding state between the grid and the ECC matrix was still sufficient.

**Figure 3:**
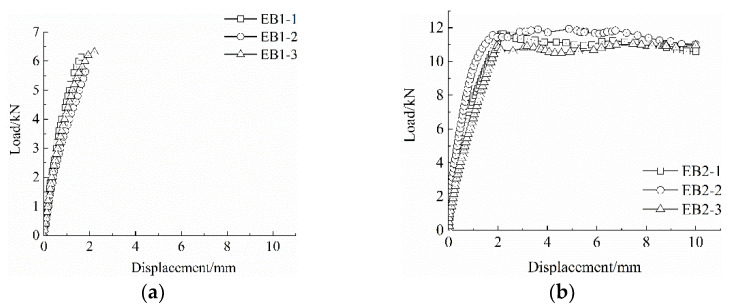

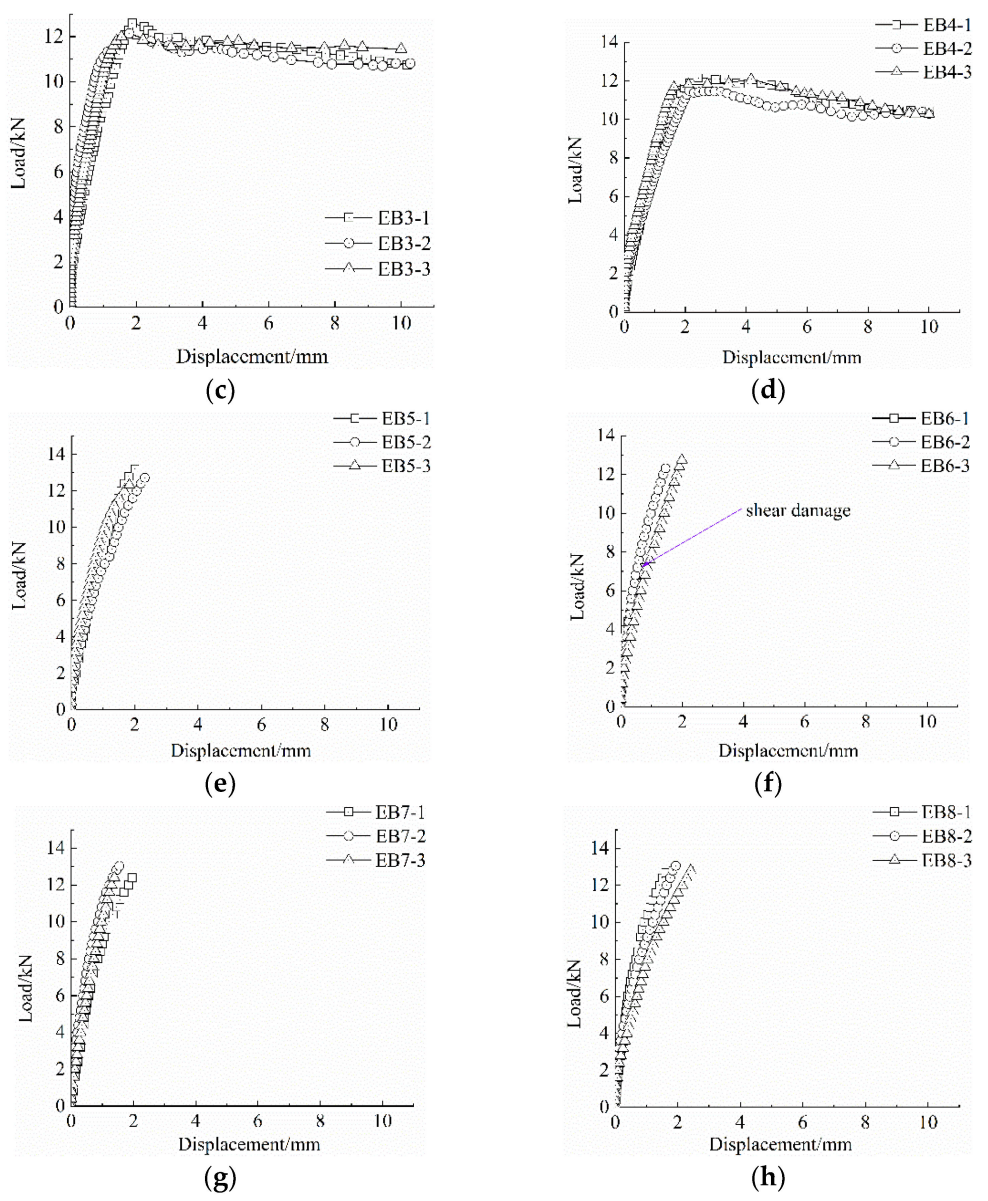
Bond–slip curve. (**a**) EB1, (**b**) EB2, (**c**) EB3, (**d**) EB4, (**e**) EB5, (**f**) EB6, (**g**) EB7, and (**h**) EB8.

The test results are shown in Table 5, and the bond–slip curve is shown in Figure 3.

It can be seen from Figure 3 that the bond–slip curve ((b) EB2, (c) EB3, (d) EB4) can be roughly divided into two stages: the first is the rising stage of non-linear growth, and the second is the roughly stable horizontal stage. At the initial stage of the curve’s rising section, only slight slippage occurs between the grid and the matrix, and the bonding force between the two is mainly supported by the chemical bonding force. As the drawing force gradually increases, the debonding zone becomes longer and the mesh slip rate increases. Until the debonding section extends to the free end, the chemical bonding force is basically lost, and the mechanical bite force and friction force provide the bonding force. After entering the residual phase, the pull-out force no longer increases and only a small range of fluctuations occur until the pull-out displacement of the grid steadily increases.

## 3. Bond–Slip Constitutive Model

### 3.1. Existing Bond–Slip Constitutive Models at Home and Abroad

1. The Malvar model [26] is suitable for situations where there is lateral pressure, or the strength of concrete is not unique.

(4) $$\tau_{m}/f_{t}=A+B\left(1-e^{-C\sigma /f_{t}}\right)$$

(5) $$S_{m}=D+E\sigma$$

(6) $$\;\tau =\tau_{m}\frac{F\left(\frac{s}{s_{m}}\right)+\left(G-1\right){\left(\frac{s}{s_{m}}\right)}^{2}}{1+\left(F-2\right)\left(\frac{s}{s_{m}}\right)+G{\left(\frac{s}{s_{m}}\right)}^{2}}$$

where $\tau_{m}$ is the peak bond stress, $S_{m}$ is the corresponding slip, σ is the axially symmetric radial pressure, and $f_{t}$ is the tensile strength of concrete. *A*, *B*, *C*, *D*, and *E* are constants determined based on test results. *F* and *G* are constants determined according to the type of FRP bar and the test results.

2. The BPE model [27] is commonly used to describe the bond–slip relationship between deformed steel bars and concrete.

(7) $$\left\{\begin{array}{l} \tau /\tau_{1}={\left(s/s_{1}\right)}^{\alpha }\;\left(s\leq s_{1}\right)\\ \tau =\tau_{1}\;\left(s_{1}\leq s\leq s_{2}\right)\\ \tau =\tau_{1}-\frac{\tau_{1}-\tau_{3}}{s_{2}-s_{3}}(s_{2}/s)\;\left(s>s_{3}\right) \end{array}\right.$$

where $\tau_{1}$ is the peak bond stress, $S_{1}$ is the corresponding slip, and α is a constant less than or equal to 1. $S_{2}$, $S_{3}$, and $\tau_{3}$ are determined according to the test results.

3. The improved BPE model [28] can be used to analyze the bond–slip relationship between FRP bars and concrete.

(8) $$\left\{\begin{array}{l} \tau /\tau_{1}={\left(s/s_{1}\right)}^{\alpha }\;\left(s\leq s_{1}\right)\\ \tau /\tau_{1}=1-p\left(s/s_{1}-1\right)\;\left(s_{1}<s<s_{3}\right)\\ \tau =\tau_{3}\;\left(s>s_{3}\right) \end{array}\right.$$

where α and p are the parameters determined based on the test results, and $\tau_{3}$ is the component of friction.

4. The CMR model [29] only considers the ascending section, which satisfies most structural calculation needs, but is not suitable for analyzing the entire force process of components.

(9) $$\tau /\tau_{m}={\left(1-e^{-s/s_{r}}\right)}^{\beta }$$

where $\tau_{m}$ is the peak bond stress, and $S_{r}$ and β are the fitting parameters obtained based on the test curve.

5. In the continuous curve model [30], the physical concept of the curve is relatively clear.

(10) $$\left\{\begin{array}{l} \tau /\tau_{1}=2\sqrt{\frac{s}{s_{0}}}-\frac{s}{s_{0}}\;\left(0\leq s\leq s_{0}\right)\;\\ \tau =\tau_{0}\frac{\left(s_{u}-s\right)2\left(2s+s_{u}-3s_{0}\right)}{{\left(s_{u}-s_{0}\right)}^{3}}+\tau_{u}\frac{\left(s-s_{0}\right)2\left(3s_{u}-2s-s_{0}\right)}{{\left(s_{u}-s_{0}\right)}^{3}}\;\left(s_{0}<s<s_{u}\right) \end{array}\right.$$

where $\tau_{0}$ and $\tau_{u}$ are the peak shear stress and residual shear stress, and $S_{0}$ and $S_{u}$ are the corresponding slip values.

### 3.2. Establishment of Constitutive Model

The bond–slip curve between the BFRP grid and the ECC has two stages: an ascending section and a horizontal section. Compared with the above models, we found that the bond–slip constitutive model between BFRP mesh and ECC is similar to the first two stages of the typical BPE model. Therefore, the constitutive model is defined as follows:

(11) $$\begin{array}{l} \frac{\tau }{\tau_{1}}={\left(\frac{s}{s_{1}}\right)}^{\alpha }\;\left(s\leq s_{1}\right)\\ \tau =\tau_{1}\;\left(s_{1}<s\leq s_{2}\right) \end{array}$$

**Figure 4:**
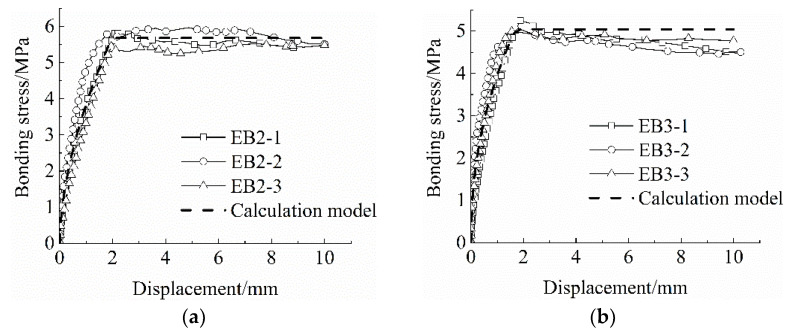

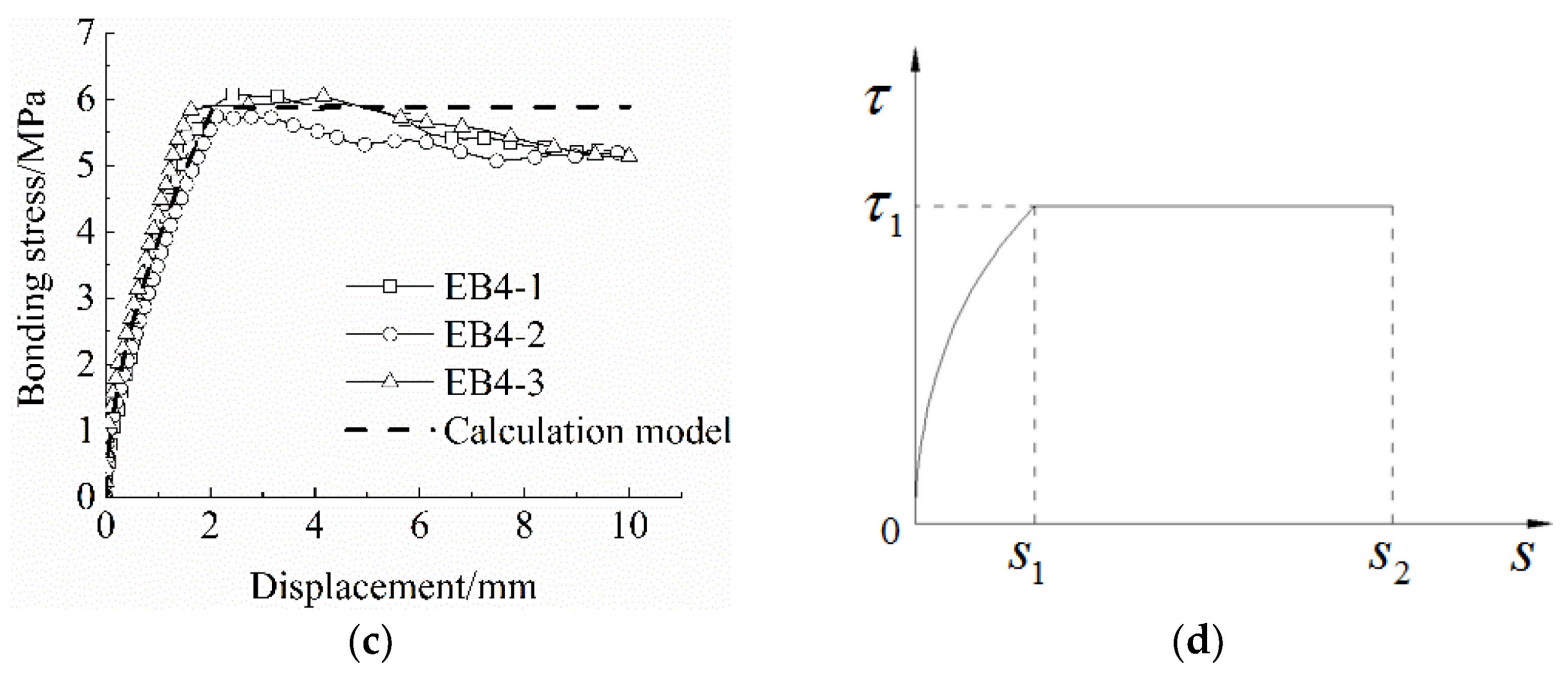
Bond–slip curve and calculation model. (**a**) EB2, (**b**) EB3, (**c**) EB4, and (**d**) bond–slip calculation model.

According to the model, the three sets of bond–slip curves were respectively fitted, and the correlation coefficients of the simplified model of the bond–slip constitutive relationship between the BFRP grid and the ECC matrix were obtained (see Table 6). The fitting correlation coefficients were all above 0.93, and the fitting results were appropriate. Figure 4 shows the fitting effect of the stick–slip curve and the calculation model. It can be seen from Figure 4 that the calculation model and the test curve are in agreement in the ascending section, and that the horizontal section has a larger error. However, since most structural analysis only considers the ascending section, and the basic anchorage length is mainly calculated based on the model ascending section, it can meet the calculation requirements of this paper.

## 4. Calculation of Anchorage Length

### 4.1. Linear Bond–Slip Model

**Figure 5:**
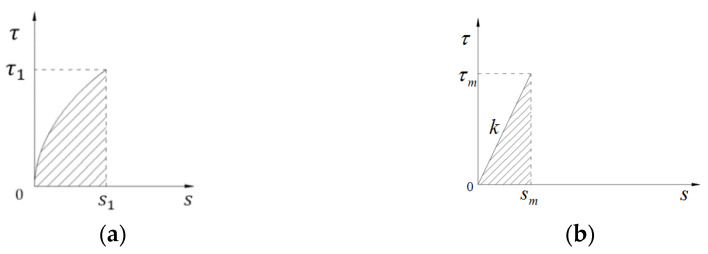
Bond–slip linear model transformation. (**a**) Actual bond–slip model and (**b**) linear bond–slip model.

According to the strain energy equivalence principle, the rising section curve of the bond–slip curve (Figure 5a) is equated to a linear bond–slip model (Figure 5b), as shown in Figure 5, where $\tau_{m}$ denotes the maximum bond force in Figure 5b, $S_{m}$ is the corresponding slip, and k is the slope of this linear model.

According to the principle of strain energy equivalence, we can obtain:

(12) $$G=\int_{0}^{s_{1}}\tau_{1}{\left(\frac{s}{s_{1}}\right)}^{\alpha }ds=\frac{\tau_{m}^{2}}{2k}$$

After sorting, we can obtain:

(13) $$k=\frac{\left(1+\alpha \right)\tau_{1}}{2s_{1}}$$

Therefore, the linear bond–slip model can be expressed as:

(14) $$\tau \left(x\right)=\frac{\left(1+\alpha \right)\tau_{1}}{2s_{1}}s\left(x\right)$$

### 4.2. Formula Derivation

Two assumptions were made in the calculation of the anchorage length: (1) that the ECC matrix and the BFRP mesh remain linearly elastic, and (2) that the tensile stresses between the BFRP mesh and the ECC matrix are uniformly distributed along the thickness direction of the matrix.

**Figure 6:**
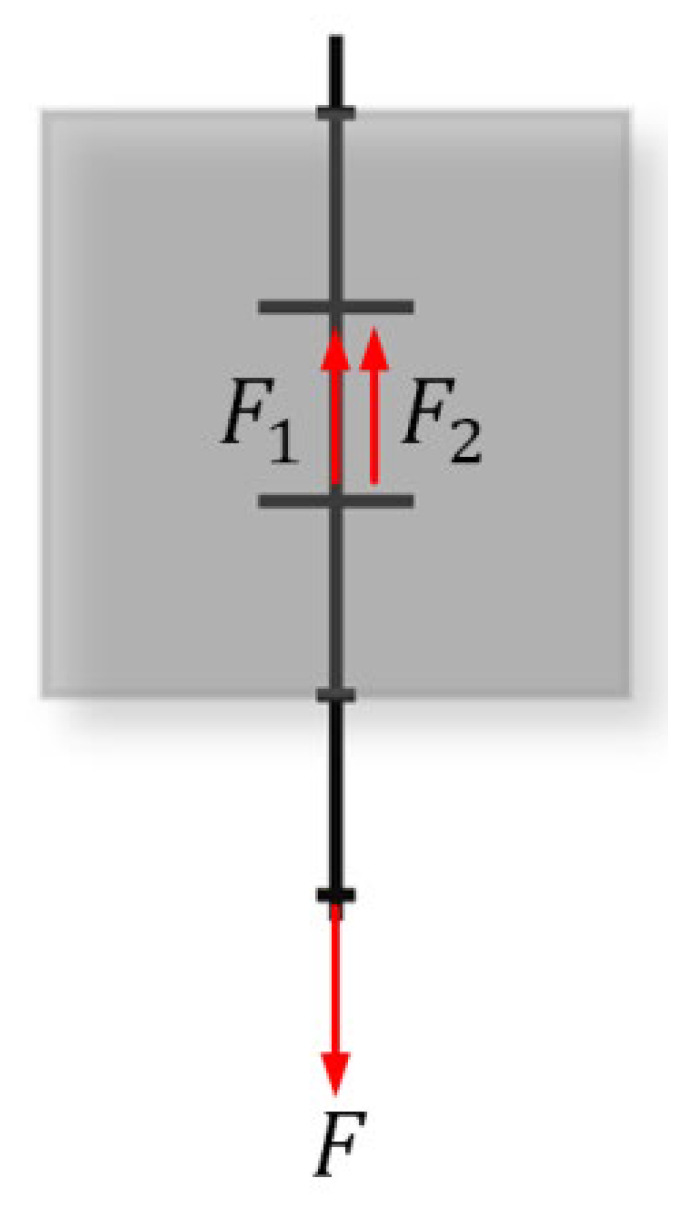
Schematic diagram of calculation of anchorage length of BFRP grid in ECC.

The calculation schematic is shown in Figure 6, where $F_{1}$ indicates the tensile force of the BFRP mesh, $F_{2}$ indicates the tensile force of the ECC, and F is a constant.

The expression for the relative slip of the BFRP grid in ECC is as follows.

(15) $$s\left(x\right)=s_{f}\left(x\right)-s_{e}\left(x\right)$$

where $s_{f}$ denotes the slip of the BFRP mesh and $s_{e}$ denotes the slip of ECC. From the tension equilibrium, it is obtained that:

(16) $$F=F_{1}\left(x\right)+F_{2}\left(x\right)$$

Taking the derivative on both sides of the equal sign yields:

(17) $$\frac{dF_{1}\left(x\right)}{dx}+\frac{dF_{2}\left(x\right)}{dx}=0$$

According to the mechanics of materials, the expression for the tensile force of BFRP mesh with ECC is:

(18) $$\left\{\begin{array}{l} F_{1}\left(x\right)=A_{f}E_{f}\frac{ds_{f}\left(x\right)}{dx}\\ F_{2}\left(x\right)=A_{e}E_{e}\frac{ds_{e}\left(x\right)}{dx} \end{array}\right.$$

where $A_{f}$ and $A_{e}$ are the cross-sectional areas of the BFRP mesh and ECC, respectively, and $E_{f}$ and $E_{e}$ denote the modulus of elasticity of the BFRP mesh and ECC, respectively. Substituting Equation (18) into Equation (17) yields:

(19) $$E_{f}A_{f}\frac{d^{2}s_{f}\left(x\right)}{dx^{2}}+E_{e}A_{e}\frac{d^{2}s_{e}\left(x\right)}{dx^{2}}=0$$

After quadratic differentiation of Equation (15) combined with Equation (19), we obtain:

(20) $$\left\{\begin{array}{l} \frac{d^{2}s_{f}\left(x\right)}{dx^{2}}=\frac{E_{e}A_{e}}{E_{f}A_{f}+E_{e}A_{e}}\frac{d^{2}s\left(x\right)}{dx^{2}}\\ \frac{d^{2}s_{e}\left(x\right)}{dx^{2}}=-\frac{E_{f}A_{f}}{E_{f}A_{f}+E_{e}A_{e}}\frac{d^{2}s\left(x\right)}{dx^{2}} \end{array}\right.$$

From the equilibrium of the micro-segments of the BFRP grid, it follows that:

(21) $$\frac{dF_{1}\left(x\right)}{dx}=u_{f}\tau \left(x\right)$$

where *A* and *B* are the section perimeter and surface shear stress of the BFRP mesh, respectively. Combining Equations (14), (18), (20), and (21) yields:

(22) $$\frac{d^{2}s\left(x\right)}{dx^{2}}=\frac{E_{f}A_{f}+E_{e}A_{e}}{E_{f}A_{f}E_{e}A_{e}}u_{f}ks\left(x\right)$$

For ease of derivation, let $\lambda =\sqrt{\frac{E_{f}A_{f}+E_{e}A_{e}}{E_{f}A_{f}E_{e}A_{e}}u_{f}k}$, then Equation (22) can be expressed as:

(23) $$\frac{d^{2}s\left(x\right)}{dx^{2}}=\lambda^{2}s\left(x\right)$$

Solving the equation yields:

(24) $$s\left(x\right)=c_{1}\exp\left(-\lambda x\right)+c_{2}\exp\left(\lambda x\right)$$

where $c_{1}$ and $c_{2}$ are constants to be determined and can be solved by the following boundary conditions:

(25) $$\left\{\begin{array}{l} \frac{ds\left(x\right)}{dx}\left|\begin{array}{l} {}_{x=0} \end{array}\right.=0\\ \frac{ds\left(x\right)}{dx}\left|\begin{array}{l} {}_{x=l} \end{array}\right.=\frac{F}{E_{f}A_{f}}-\frac{F}{E_{e}A_{e}} \end{array}\right.$$

Solving the equation yields:

(26) $$c_{1}=c_{2}=\frac{\left(\frac{F}{E_{f}A_{f}}-\frac{F}{E_{e}A_{e}}\right)}{\lambda \left[\exp\left(\lambda l\right)-\exp\left(-\lambda l\right)\right]}$$

Therefore, Equation (24) can be simplified as:

(27) $$s\left(x\right)=\frac{1}{\lambda }\frac{\exp\left(\lambda x\right)+\exp\left(-\lambda x\right)}{\left[\exp\left(\lambda x\right)-\exp\left(-\lambda x\right)\right]}\left(\frac{F}{E_{f}A_{f}}-\frac{F}{E_{e}A_{e}}\right)$$

The expression for the pullout force *A* can be obtained from Equation (27) as follows:

(28) $$F\left(x\right)=\lambda s\left(x\right)\cdot \frac{\exp\left(\lambda x\right)-\exp\left(-\lambda x\right)}{\left[\exp\left(\lambda x\right)+\exp\left(-\lambda x\right)\right]}\cdot \frac{E_{f}A_{f}E_{e}A_{e}}{E_{e}A_{e}-E_{f}A_{f}}$$

when $s\left(x\right)=s_{m}$, the BFRP grid reaches the ultimate load, $F_{m}$:

(29) $$F_{m}=\lambda s_{m}\tanh\left(\lambda l_{ab}\right)\frac{E_{f}A_{f}E_{e}A_{e}}{E_{e}A_{e}-E_{f}A_{f}}$$

The basic anchorage length, $l_{ab}$, of the BFRP mesh in the ECC matrix is obtained from Equation (29) as:

(30) $$l_{ab}=\frac{1}{2\lambda }\ln\left[\frac{\lambda s_{m}E_{f}A_{f}E_{e}A_{e}+F_{m}\left(E_{e}A_{e}-E_{f}A_{f}\right)}{\lambda s_{m}E_{f}A_{f}E_{e}A_{e}-F_{m}\left(E_{e}A_{e}-E_{f}A_{f}\right)}\right]$$

### 4.3. Formula Verification

The basic anchorage lengths of the test piece groups, whose grids were pulled out, were calculated from Equation (30), as shown in Table 7.

The actual anchorage lengths under the same test conditions were 100 mm (EB2) and 150 mm (EB6) when the specimens were damaged by mesh pullout and mesh fracture, respectively. Therefore, the calculated basic anchorage length should be at least between 100 and 150 mm to have certain reliability. Comparing the calculated length with the actual length, it can be seen that the calculated length is within the tolerance range and the calculation result has some reliability. Considering the requirements of engineering safety and material variability, the proposed values of basic anchorage lengths for each specimen are shown in Table 8.

## 5. Conclusions

In this study, we conducted bond–slip tests on eight sets of BFRP mesh pullout specimens and established a bond–slip intrinsic model between the BFRP mesh and ECC based on the bond–slip curve characteristics of the specimens. Combined with the proposed intrinsic relationship model, the basic anchorage length calculation formula between BFRP mesh and ECC was derived based on the strain energy equivalence principle. The main conclusions are as follows.

(1) The bonding performance between the BFRP mesh and ECC improves with the increase in the anchorage length of the mesh, thickness of the mesh, and strength of the ECC layer, and the bonding force can be enhanced by sticking sand on the surface of the BFRP mesh. The bond–slip curve can be divided into two stages: the rising stage with non-linear growth and the horizontal stage with roughly smooth growth.
(2) The bond–slip relationship between the BFRP mesh and ECC can be described by the first two stages in the BPE model.
(3) The derived formula for calculating the basic anchorage length of the BFRP mesh in the ECC matrix is computationally verified to be reliable in prediction. The suggested values of basic anchorage length for each specimen were conservatively proposed according to the variability of materials.
(4) The bonding performance between the BFRP mesh and ECC studied in this paper was developed based on the average bonding force, while in fact the stress distribution of the BFRP mesh in ECC along the anchorage length of the mesh was not uniform. Further studies are still needed to verify the stress distribution pattern of the BFRP mesh in ECC along the anchorage length.

## Figures and Tables

**Table 1:** ECC mix ratio (quality ratio).

Specimen	Ordinary Silicate Cement	Water	Fly Ash	Silica Fume	Quartz Sand	Water-Reducing Agent	PVA Volume Rate (%)
ECC1	1	0.9	1.5	0.05	1	0.014	2
ECC2	1	0.5	0.4	0.05	0.36	0.007	2

**Table 2:** Performance index of PVA fiber.

Length/mm	Diameter/μm	Density/(g/cm^3^)	Tensile Strength/MPa	Modulus of Elasticity/GPa	Elongation/%
12	39	1.3	1620	42.8	7

**Table 3:** BFRP grid material performance indicators.

Thickness of Mesh/mm	Average Tensile Strength/MPa	Modulus of Elasticity/GPa	Post-Break Extension Rate/%
2 mm	468	51.7	2.72
3 mm	495	54.5	2.46
5 mm	522	58.1	2.07

**Table 4:** Specimen design.

Specimen	ECC	Thickness of the Grid/mm	Anchorage Length/mm	Protective Layer Thickness/mm	Surface Treatment
EB1	ECC1	2	100	15	/
EB2	ECC1	3	100	15	/
EB3	ECC1	5	100	15	/
EB4	ECC2	3	100	15	/
EB5	ECC1	3	100	20	/
EB6	ECC1	3	150	15	/
EB7	ECC1	3	200	15	/
EB8	ECC1	3	100	15	Stick the sand

**Table 5:** The main test results of each drawing specimen set.

Specimen	Average Ultimate Load/kN	Average Bond Strength/MPa	Main Damage Types
EB1	6.02	/	The grid was broken
EB2	11.36	5.68	The grid was pulled out
EB3	12.25	5.10	The grid was pulled out
EB4	11.76	5.88	The grid was pulled out
EB5	12.76	/	The grid was broken
EB6	12.51	/	The grid was broken
EB7	12.70	/	The grid was broken
EB8	12.95	/	The grid was broken

**Table 6:** Curve constants and fitting coefficients of the specimen group.

Specimen	$\tau_{1}$	$s_{1}$	α	$R^{2}$
EB2	5.68	1.97	0.57875	0.94
EB3	5.10	1.74	0.45793	0.93
EB4	5.88	2.06	0.58133	0.96

**Table 7:** Calculated anchorage length of the BFRP grid in the ECC matrix.

Specimen	$\mathrm{Anchorage}\;\mathrm{Length},\;l_{\mathrm{ab}}/\mathrm{mm}$
EB2	134
EB3	178
EB4	128

**Table 8:** Recommended basic anchorage lengths of the BFRP grid in the ECC matrix.

Specimen	$\mathrm{Anchorage}\;\mathrm{Length},\;l_{\mathrm{ab}}/\mathrm{mm}$
EB1	150
EB2	200
EB3	250
EB4	200
EB5	200
EB6	200
EB7	200
EB8	200

## Data Availability

The data presented in this study are available upon request from the corresponding author.
